# Experience of the new seizure diary in the Free State and Northern Cape

**DOI:** 10.4102/safp.v65i1.5736

**Published:** 2023-05-26

**Authors:** Chika K. Egenasi, Anandan A. Moodley, Wilhelm J. Steinberg, Gina Joubert

**Affiliations:** 1 Department of Family Medicine, School of Clinical Medicine, Faculty of Health Sciences, University of the Free State, Bloemfontein, South Africa; 2 Department of Neurology, Faculty of Health Sciences, University of KwaZulu-Natal, Durban, South Africa; 3 Department of Biostatistics, School of Biomedical Sciences, Faculty of Health Sciences, University of the Free State, Bloemfontein, South Africa

**Keywords:** seizure diary, epilepsy, paper diary, electronic diary, seizure frequency, participants who had previous exposure to a seizure diary, previously diary-unexposed participants

## Abstract

**Background:**

Epilepsy is a neurological disease affecting adults and children globally. A seizure diary is one of the self-management tools for tracking seizures. This study aims to ascertain the experience of a new seizure diary by persons completing the diary in the Free State and Northern Cape of South Africa.

**Methods:**

Adult patients with epilepsy attending Universitas Academic Hospital epilepsy clinic in Bloemfontein, clinics in Kimberley and the casualty department of Kimberley hospital (Robert Mangaliso Sobukwe hospital) received a new seizure diary. After using the diary for 6 months, participants (patients, relatives or caregivers) completed a questionnaire.

**Results:**

A total of 139 epilepsy patients received a new seizure diary; 67 previously diary-unexposed participants and 33 participants who had previous exposure to a seizure diary. The majority of participants, namely 91% of previously diary-unexposed and 84.9% of participants who had previous exposure to the seizure diary, understood the new seizure diary. Participants who had previous exposure to a seizure diary were predominantly very positive about the new diary because it had more information. However, 21.2% indicated that they preferred the old one because it was easier to complete.

**Conclusion:**

Patients, caregivers or relatives from both groups used the new seizure diary and provided important information about their experience with the new diary. Despite a few complaints about using the new diary, most participants who had previous exposure to a seizure diary preferred the new seizure diary.

**Contribution:**

This study explored participants’ opinions of the new seizure diary.

## Introduction

Epilepsy is common and one of the most dynamic disorders in neurology.^1^ It affects millions of people worldwide, of which about 80% live in low- and middle-income countries.^2^ Epilepsy contributes about 0.5% of the global disease burden.^3^ Globally, about 5 million people are diagnosed with epilepsy each year. It is estimated that in high-income countries, 49 per 100 000 people are diagnosed with epilepsy yearly; in low- and middle-income countries, it can be as high as 139 per 100 000.^2^ In South Africa, the prevalence of active convulsive epilepsy in rural areas was reported as 7 per 1000.^4^ Treatment gaps exist in South Africa, as in most low-income countries, and anti-seizure medication adherence remains low despite adequate diagnosis and access to healthcare facilities.^5^ Factors such as lack of adequately trained primary healthcare practitioners, neurologists, healthcare facilities, poor medication adherence and socioeconomic and cultural beliefs may adversely affect epilepsy treatment.^3,6^ Furthermore, epilepsy is poorly understood, and the different cultural understandings and terms used to explain the condition across the various population groups compound the problems of these patients.^7^ The management of epilepsy in our environment must aim to control the seizures to reduce morbidity and mortality associated with epilepsy.^8^

Seizure types and frequencies are important outcomes reported in epilepsy clinical care and research.^9^ Seizure documentation is an important aspect of care for patients living with epilepsy; in certain situations, patients may be unable to document their seizures because of being seizure free, so they have no seizures to document or other reasons.^9^ If patients with epilepsy know when and how their seizures occur, it can aid in diagnosis and treatment.^10^ A seizure diary is a self-management tool designed to help patients with epilepsy track their seizures and side effects, manage anti-seizure medications and see their progress over time.^11^ It can be used in clinical drug trials, monitoring seizure clusters and predicting seizures.^12,13,14,15,16,17,18^

Diaries are known to be either paper based or electronic diaries. The paper diary is a basic calendar with seizure types and frequency, which is the industry standard.^18^ Paper diaries have historically been used to document the seizure frequencies of patients with epilepsy.^10^ Inherent inadequacies have been reported with paper diaries, such as being easy to misplace, lose or damage.^10,19^ On the other hand, electronic diaries are online or mobile applications with more details and time-stamped patient entries available to patients, caregivers (persons caring for the chronically ill, elderly or children), relatives (members of the family) and healthcare workers.^18^ They can be downloaded from android (Google play) and IOS (Apple mobile operating system) application stores. The electronic diary can also send reminders to patients to take their medications or log their seizures and are available in different languages.^10,20^

In our previous publications of this study,^21,22^ we discussed the advantages of the paper-based seizure diary in that it does not require Internet access or expensive devices; it is easier for patients to learn how to use; it is less complex because it does not need great intellectual capabilities or specialised computer skills to complete. In addition, a paper-based diary can be successfully used by a predominantly illiterate community and can easily be made available to patients.^23^ Accurate seizure documentation entails patients and caregivers recognising their seizures and being compliant with keeping a seizure diary.^9^ Some of the drawbacks of the seizure diary are the under-reporting or over-reporting of self-reported seizure counts by patients and caregivers because of a lack of awareness.^12,24,25,26^

In South Africa, there is no documented standard format for the seizure diary. A calendar format of the diary, designed by pharmaceutical companies, is used in some South African medical facilities,^27^ such as the Bloemfontein neurology clinic. Other available formats are intended for patient use in western countries such as the United Kingdom.^28^ An extensive literature review has shown that a properly designed seizure diary is still relevant in managing patients with epilepsy if they are adequately trained to use it.^21,29^ Motivating the patients and their caregivers to use the diary will further help improve compliance with diary usage.^21^ Patients can use this opportunity to reflect on their seizure experiences by commenting on the diary, thereby giving the healthcare provider a window of opportunity to understand the disease from the patient’s view more holistically. Clinicians should manage their patients holistically based on the principles of patient-centred care using the biopsychosocial approach.^30^

The development of new patient diaries, electronic applications and mobile software requires the input of patients living with epilepsy and caregivers to make them more effective in documenting and managing the disease.^9^ The user experience of seizure management tools can help to gauge patients’ satisfaction,^31^ and the data obtained can be helpful in future epilepsy research.^9^

Our study aimed to gather information on the experience of patients living with epilepsy with a new seizure diary in the Free State and Northern Cape of South Africa. The new seizure diary was developed based on expert opinions and patient inputs about the contents of a seizure diary for monitoring and managing patients living with epilepsy. The new diary had more detailed information, such as seizure types, frequencies, duration, current seizure medications and other information.^32^

## Research methods and design

### Study design

This study was part of a more extensive study with five phases, some of which have been published.^21,22^ Earlier phases consisted of Phase 1 (Scoping review of literature),^21^ Phase 2 (The perceptions and attitudes of patients with epilepsy to the use of a seizure diary in managing patients with epilepsy),^22^ and Phase 3 (Delphi study).^32^ This manuscript reports on Phase 4, a longitudinal study (Use of the new, improved seizure diary for 6 months), 5a, a cross-sectional study (Experience of the new seizure diary) and 5b (Suggested final version of the seizure diary).

### Study population

The study population consisted of adults with epilepsy in Kimberley and Bloemfontein, currently attending Universitas Academic Hospital Specialist Epilepsy Clinic in Bloemfontein and local clinics in Kimberley (City, Beaconsfield and Betty Gatsewe), as well as the casualty department in Kimberley hospital (Robert Mangaliso Sobukwe Hospital). Participants in the study were patients, relatives and caregivers who assisted in completing the diary. They also assisted with completing the questionnaires. Patients could be previously (before the study) diary-unexposed or have had previous exposure to a seizure diary.

### Inclusion criteria

Patients diagnosed with epilepsy attending the facility for follow-up, 18 years and above, were recruited for the study. The patient must have participated in Phase 2 of the study and received the new seizure diary. Patients with cognitive impairment were included as relatives provided information. Informed consent was obtained from patients or caregivers when patients were disabled owing to cognitive impairment.

### Sampling method

All patients who had taken part in Phase 2 of the study were included.

### Sample size

The intended sample size for the study was 182 patients (117 previously diary-unexposed and 65 who had previous exposure to a seizure diary).^32^ No formal sample size calculation was performed, and this study had no main outcome on which to base such a calculation, but this group size gives sufficient power (80%) to detect large differences between the groups and fairly precise estimates within groups. However, once the new diary was designed, only 139 patients (81 and 58 patients, respectively) could be traced to receive and use the new diary.

### Measurement

We used a confidential, structured questionnaire with 22 open- and closed-ended questions. It explored the persons’ experience of using the new seizure diary and consisted of two sections A and B.

Section A consisted of seven demographic questions about the person responsible for completing the diary. Section B had 15 questions about the participants’ use of the new seizure diary, requiring a ‘Yes’, ‘No’ or ‘Not Sure’ response and a question exploring the reasons for not understanding the diary. Further questions were asked when the diary was completed, where the diary was kept, if the patients want to continue using the diary and what they want to be improved.

Participants were asked if they had previously used a seizure diary; if the answer was yes, they were required to proceed to the last question and if no, they had completed the survey. The last question required the participants to choose their preferred diary and explain why. The questionnaires were available in English, Afrikaans, Setswana and Sesotho. Two research assistants with healthcare experience were trained to assist the first author. They could communicate in English, Afrikaans, Setswana or Sesotho and were willing to do home visits in Kimberley and Bloemfontein.

The first author or the assistants tracked and manually distributed the questionnaire in City clinic, Betty Gatsewe, Beaconsfield Clinic, Robert Mangaliso Sobukwe Hospital casualty and Bloemfontein neurology clinic while patients were in the waiting area waiting to be called to see the doctor. The first author and his assistants explained to the patients and caregivers that this was a follow-up on the diary they were using and obtained informed consent from the participants. All participants were required to complete the questionnaires and return the completed questionnaire to the researcher or his assistants while still in the waiting area. Patients were assisted by their relatives, caregivers, the researcher or trained assistants fluent in one of the spoken languages. Cognitively impaired patients were assisted by their relatives or caregivers. As a result of patients not attending the clinics monthly since the coronavirus disease 2019 (COVID-19) pandemic, the number of patients found in the clinic was relatively low. The first author and research assistants repeatedly visited the homes of many patients not found during previous visits. They also tried to locate the patients by asking the neighbours to inform them of their visits and leaving their contact details.

### Pilot study

The questionnaire was piloted on epilepsy patients in Kimberley Hospital casualty and Universitas Academic Hospital neurology specialist clinic. It included four patients who received the new diary. This tested the questionnaire and the project processes. Data from the pilot study were not used in the main study because of changes in the questionnaire to further clarify who is completing the seizure diary.

### Statistical analysis

Categorical variables were summarised by frequencies and percentages and numerical variables by median and interquartile ranges (IQRs) because of skewed distributions. Groups were compared regarding categorical variables using chi-square or Fisher’s exact tests (in the case of sparse cells). The significance level was set at 0.05. Analysis was performed using SAS^®^ Version 9.4.

### Ethical considerations

The amended protocol for the study was approved by the Health Sciences Research Ethics committee (HSREC) of the University of the Free State with reference number UFS-HSD2020/1385/2411. Approval for data collection was also obtained from the Northern Cape Health Department and the Free State Health Department.

Number coding was used to ensure the confidentiality of the participant’s responses. No names or personal identifiers appeared on any research-related information or datasheet sent for statistical analysis. The researcher kept all paper-based records in a secure location, and these records were only accessible to those involved in the study. All information was managed in a confidential manner.

## Results

A total of 139 patients (81 previously diary-unexposed and 58 who had previous exposure to a seizure diary) received and used the diary for 6 months, after which they were asked to complete a questionnaire from August 2022 to November 2022. Responses of 100 participants (67 previously diary-unexposed and 33 who had previous exposure) were available. There was a dropout rate of 28.1% because of multiple factors such as change of address, change of phone numbers or missed appointments. Eleven patients were seizure free and could not complete the diary, and five patients died after receiving the seizure diary (see Figure 1).

**FIGURE 1:**
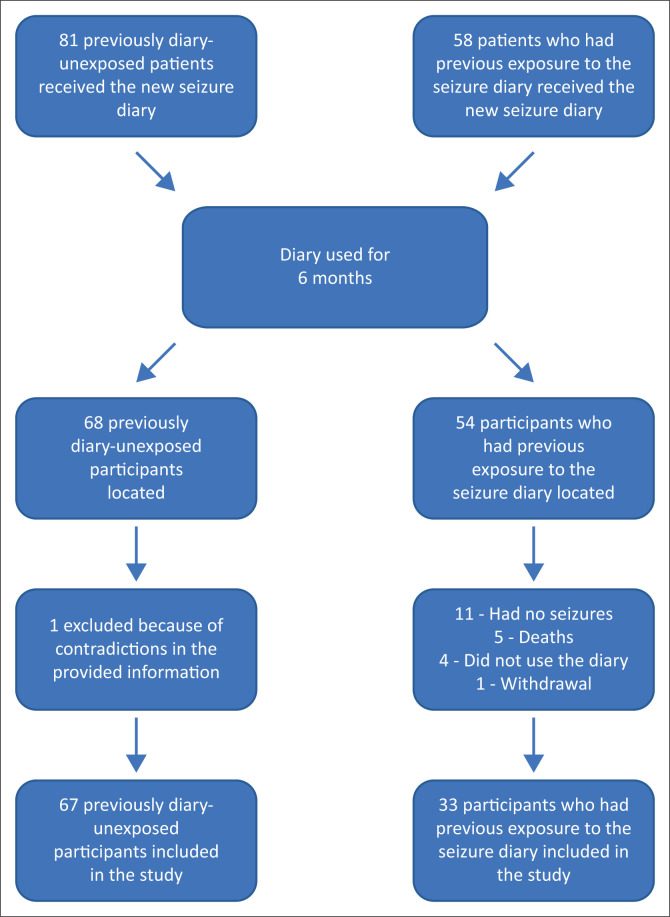
A flow diagram of participantsin the study.

The median age for patients, caregivers or relatives completing the questionnaire was 45 years (range: 19–76 years) in the diary-unexposed group and 41 years (range 24–75 years) in the participants who had previous exposure to the seizure diary (*p* = 0.463).

Table 1 indicates that most of the persons completing the diary were females, unemployed and had below Grade 12 education in both study groups. A statistically significant difference was found between previously diary-unexposed participants and participants who had previous exposure to a seizure diary regarding gender and educational qualifications.

**TABLE 1:** Demographic characteristics of persons completing the seizure diary.

Variables	Previously diary-unexposed		Participants who had previous exposure to a seizure diary		*p*
	*n* †	%	*n* ‡	%	
**Gender**	-	-	-	-	0.015
Male	18	26.9	2	6.1	-
Female	49	73.1	31	93.9	-
**Occupation**	-	-	-	-	0.809
Employed	14	20.9	5	15.2	-
Pensioner	13	19.4	6	18.2	-
Student	2	3.0	2	6.1	-
Unemployed	38	56.7	20	60.6	-
**Educational qualification**	-	-	-	-	0.012
Below Grade 12	38	56.7	18	54.6	-
Matric	25	37.3	6	18.2	-
University	3	4.5	5	15.2	-
Others	1	1.5	4	12.1	-

† , *n* = 67;

‡ , *n* = 33.

Figure 2 shows the distribution of participants completing the diary. A small number of patients completed the diary in the previously diary-unexposed and participants who had previous exposure to a seizure diary group. No significant differences were observed between the two groups (*p* = 0.244).

**FIGURE 2:**
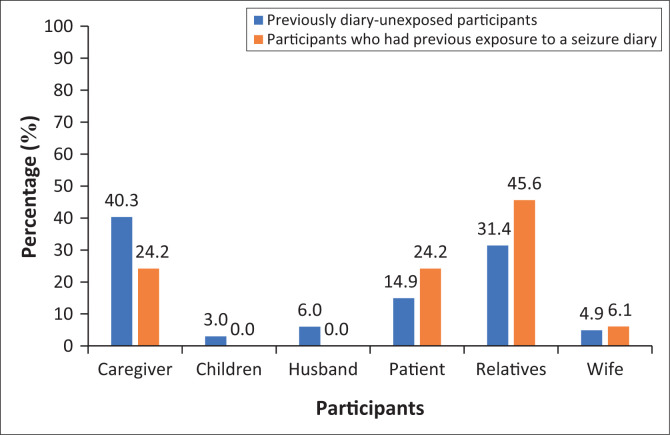
Persons completing the new seizure diary.

Table 2 and Table 3 show that almost all persons completing the diary found the new seizure diary to be useful in managing their epilepsy. No significant differences were found between previously diary-unexposed participants and participants who had previous exposure to a seizure diary except for the size of the new diary (*p* = 0.010).

**TABLE 2:** Use of the seizure diary: Part 1.

Questions about the use of the new seizure diary	Previously diary-unexposed participants(*n* = 67)						Participants who had previous exposure to a seizure diary (*n* = 33)					
	Yes		No		Not sure		Yes		No		Not sure	
	*n*	%	*n*	%	*n*	%	*n*	%	*n*	%	*n*	%
Do you understand the new seizure diary?	61	91.0	4	6.0	2	3.0	28	84.9	2	6.1	3	9.1
Do you like the size of the new seizure diary?	67	100.0	0	0.0	0	0.0	29	87.9	4	12.1	0	0.0
Do you think the new seizure diary is useful?	66	98.5	1	1.5	0	0.0	33	100	0	0.0	0	0.0
Do you enjoy completing the new seizure diary?	64	95.2	1	1.5	2	3.0	32	97.0	0	0.0	1	3.0
Does the new seizure diary help track your/patient’s seizures?	67	100.0	0	0.0	0	0.0	33	100.0	0	0.0	0	0.0
Does the new diary help relatives/caregivers keep track of your/patient’s seizures?	67	100.0	0	0.0	0	0.0	33	100.0	0	0.0	0	0.0
Do you find the new diary useful in helping to provide information about your/patient’s current medications?	64	95.5	2	3.0	1	1.5	33	100.0	0	0.0	0	0.0
Does the new diary help to provide information when you meet with your/patient’s doctor?	65	97.0	1	1.5	1	1.5	33	100.0	0	0.0	0	0.0
Do you want to continue using the new seizure diary in future?	66	98.5	1	1.5	0	0.0	32	97.0	1	3.0	0	0.0
Do you think anything should change in order to further improve the new diary?	1	1.5	66	98.5	0	0.0	5	15.2	28	84.9	0	0.0

**TABLE 3:** Use of the seizure diary: Part 2.

Questions about the use of the new seizure diary	Previously diary-unexposed participants (*n* = 67)		Participants who had previous exposure to the seizure diary (*n* = 33)	
	*n*	%	*n*	%
**After a fit, when do you complete the new diary?**				
Immediately	51	76.1	27	81.8
1 day later	13	19.4	5	15.2
2 days to ≤ a month	3	4.5	1	3.0
1–2 months	0	0.0	0	0.0
3–5 months	0	0.0	0	0.0
≥ 6 months	0	0.0	0	0.0
**Where do you keep the new seizure diary?**				
Handbag	21	31.3	11	33.3
In the drawer	17	25.4	14	42.4
On the table	15	22.4	7	21.2
On the fridge	3	4.5	0	0.0
In the office	1	1.5	0	0.0
**Others**				
File	5	7.5	0	0.0
Bible	1	1.5	0	0.0
Medication bag	1	1.5	0	0.0
Car	1	1.5	0	0.0
Under the mattress	1	1.5	0	0.0
Wardrobe	1	1.5	1	3.0

Note: Why do you think the new seizure diary is useful? – previously diary-unexposed participants *n* = 66; participants who previously used a seizure diary *n* = 33.

Six previously diary-unexposed participants and five participants who had previous exposure to the seizure diary reported that they did not understand the diary (see Table 2 and Table 3). Of those participants previously diary-unexposed, three (50%) said, ‘it was too confusing for me’, two (33.3%) previously diary-unexposed and one (20%) participant who had previous exposure to the seizure diary said, ‘How to use it was not explained to me’, another one (20%) participant who had previous exposure to the seizure diary and one (16.7%) previously diary-unexposed participant stated, ‘I don’t know what to do with it’. Two participants who had previous exposure to the seizure diary (40%) reported, ‘I find it too difficult to understand’, and one reported, ‘The language was too difficult to understand’ (20%).

Table 4 shows that most of the participants from both groups were satisfied with the size of the new seizure diary because it is easy to carry around. A few participants who had previous exposure to a seizure diary (*n* = 4; 12.1%) reported that they did not like the size of the new seizure diary because ‘It was too small to record in full’ (*n* = 2; 50%), ‘would like it to be bigger’ (*n* = 1; 25%), and ‘it was too long’(*n* = 1; 25%).

**TABLE 4:** Reasons explaining the response to questions about the use of the diary.

Reasons categorised	Previously diary-unexposed participants		Participants who previously used a seizure diary	
	*n*	%	*n*	%
**Why do you like the size of the new seizure diary**?				
Easy to carry around/hold	52	77.6	16	55.2
Fits my handbag	8	11.9	8	27.6
Like the size	4	6.0	1	3.4
Others	3	4.5	4	13.8
**Why do you think the new seizure diary is useful?**				
Helps to track seizure	55	83.3	26	78.8
Provides information	5	7.6	4	12.1
Helps Drs keep record	4	6.1	2	6.1
Others	2	3.0	1	3.0
**Why do you enjoy completing the new seizure diary?**				
Helps family monitor seizures	25	39.0	19	59.4
Provides information about seizures	30	46.9	10	31.3
Helps Drs understand the seizures	3	4.7	-	-
Easier to use	6	9.4	3	9.4
Others	0	0.0	0	0.0

Note: Why do you like the size of the new seizure diary? – previously diary-unexposed participants *n* = 67; participants who previously used a seizure diary *n* = 29. Why do you think the new seizure diary is useful? – previously diary-unexposed participants *n* = 66; participants who previously used a seizure diary *n* = 33.

Some of the previously diary-unexposed participants and participants who had previous exposure to a seizure diary requested changes to the new seizure diary (see Table 2 and Table 3). The changes requested by previously diary-unexposed include ‘space for management after an attack’ (*n* = 1; 100%), while participants who had previous exposure to a seizure diary requested ‘extra columns for remarks’ (*n* = 1; 20%), ‘want the diary to be available in the Afrikaans language’ (*n* = 1; 20%), and ‘explain the types of seizures better’ (*n* = 3; 60%).

Only the participants who had previous exposure to a seizure diary were asked to compare the previous diary to the new seizure diary. The majority, (*n* = 26; 78.8%, 95% confidence interval [CI]: 61.1% to 91.0%), preferred the new seizure diary, while 7 (21.2%) preferred the old seizure diary. A summary of the reasons is represented in Table 5.

**TABLE 5:** Preferred seizure diary (*n* = 33).

Participants who had previous exposure to a seizure diary	*n*	%
**Prefer new seizure diary: reasons†**		
More information	18	69.2
A better understanding of the new diary	3	11.5
More detailed diary	3	11.5
More space	1	3.8
Documents multiple seizures	1	3.8
**Prefer old seizure diary: reasons** ‡		
Easier to use	6	86.0
More useful	1	14.0

† , *n* = 26 (78.8%);

‡ , *n* = 7 (21.2%).

There was no association between the type of person completing the diary and whether they indicated that they understood the diary (Table 6 and Table 7, previously diary-exposed participants *p* = 0.828, participants who had previous exposure to a seizure diary *p* = 0.810). There was also no association between where the person keeps the diary and when the diary is completed after a seizure in previously diary-unexposed (*p* = 0.407) and participants who had previous exposure to a seizure diary (*p* = 0.515).

**TABLE 6:** Associations between responses to questions: Part 1.

Responses	Previously diary-unexposed participants (*n* = 67)			Participants who had previous exposure to a seizure diary (*n* = 33)		
	Frequency	%	*p*	Frequency	%	*p*
**Type of persons completing the diary indicating that they understood the diary**	-	-	**0.828**	**-**	**-**	**0.810**
Caregivers	23	85.2	-	8	100.0	-
Children	2	100.0	-	0	0.0	-
Husband	4	100.0	-	0	0.0	-
Patient	9	90.0	-	6	75.0	-
Relatives	20	95.2	-	12	80.0	-
Wife	3	100.0	-	2	100.0	-

**TABLE 7:** Associations between responses to questions: Part 2.

Responses	Previously diary-unexposed participants (*n* = 67)							Participants who had previous exposure to a seizure diary (*n* = 33)						
	Frequency (%)						*p*	Frequency (%)						*p*
	Immediately		1 day later		2 days to ≤ 1 month			Immediately		1 day later		2 days to ≤ 1 month		
	*n*	%	*n*	%	*n*	%	*n*	%	*n*	%	*n*	%
**Where patients keep their diary when compared with when they complete the seizure diary after a seizure**	-	-	-	-	-	-	**0.407**	-	-	-	-	-	-	**0.515**
In a handbag	18	42.9	3	25.0	0	-	-	8	30.8	2	40.0	1	100.0	-
In the drawer	9	21.4	6	50.0	2	66.7	-	11	42.3	3	60.0	0	-	-
In the office	1	2.4	0	-	0	-	-	0	-	0	-	0	-	-
On the fridge	3	7.1	0	-	0	-	-	0	-	0	-	0	-	-
On the table	11	26.2	3	25.0	1	33.3	-	7	26.9	0	-	0	-	-

## Discussion

In this study, we explored how participants (patients, relatives and caregivers) in Bloemfontein and Kimberley experienced the new seizure diary after using the diary for 6 months. Differences were found in the demographic characteristics of persons completing the seizure diary, such as more females were involved in completing the new diary than males. This may be because of the dominant roles females play in caring for sick family members; many studies support the assertion that more females are involved in playing the role of caregivers in the family.^33,34,35^ Pokharel et al. reported a female-to-male ratio of 2.5:1.^34^

Only a few patients with epilepsy could complete the new seizure diary themselves. Most often, relatives and caregivers were responsible for regularly documenting the seizures in the diary. This is in contrast with reports from other studies on patient-reported seizures. Blachut et al., in a study about patient-reported seizure counts, showed that of the 104 patients who reported keeping a seizure diary, 78% of patients reported completing their seizure diary themselves.^36^ Most of our patients may have required assistance completing their diaries because of the brief postictal state characterised by confusion and disorientation after their seizures.^37^ Most of the participants in both groups in this study (including patients) understood the new seizure diary. Fisher et al. reported that patients might be unable to understand or complete the seizure diary.^18^ The few participants who reported not understanding the diary gave reasons that point to poor communication and language barriers. This is similar to findings by Keikelame et al., who reported that communication difficulties, poor doctor–patient relationships and language barriers were perceived by doctors to affect epilepsy patient management in the primary care setting in Cape Town.^38^ The education of these patients about the seizure diary was inadequate.^27^

All participants who were previously diary-unexposed and a majority who had previous exposure to a seizure diary reported that they liked the size of the new seizure diary mainly because it was easy to hold or carry around. Fisher et al. reported that requesting too much information may make the diary impractical to use; the diary must have an efficient and user-friendly design.^18^ All the participants who had previous exposure to a seizure diary and most of the previously diary-unexposed participants reported that the new seizure diary was useful for them. The main reasons given were that it ‘helped to track seizures’, ‘provided information’, and ‘helps Drs to keep records’. These were similar to findings reported by Blachut et al. in their studies, as the main reasons stated by patients with epilepsy for documenting their seizures.^25,36^ Most participants in this study enjoyed completing the new seizure diary because it helped their families to monitor their seizures, provided more seizure information and other reasons. This was supported by reports from Blachut et al. stating that patients document seizures if they feel it is important.^25,36^ About 50% of diary documenters in that study reported that the diary provided valuable information for the doctors’ treatment decision-making and 80% found the diary easy to use.^36^

Most persons from both groups in this study completed the new seizure diary immediately after their seizures. Seizure documentation behaviour plays a significant role when it comes to underreporting seizures. The main reason for the non-documentation of observed seizures was a failure to complete the diary immediately and later forgetting.^25,36^ Many previously diary-unexposed persons kept their new seizure diary in their handbags, making it easy to carry them around. In contrast, most persons who previously used the seizure diary kept their diaries in a drawer at home. Fisher et al. reported that the seizure diary may not be at hand after a seizure event.^18^ Patients with epilepsy must be encouraged to keep the diary in a place where caregivers and family members can easily have access to the diary.

Almost all the participants from both groups were willing to continue to use the new seizure diary in future. This was similar to findings reported in our previous study on the seizure diary.^22^ The majority of participants from both groups were happy with the current format of the new seizure diary; however, a few had challenges with the new diary and wanted changes made, such as an extra column for remarks after a seizure, types of seizure explained and the diary being available in the Afrikaans language. Healthcare workers provide care for patients from different ethnic, cultural and socioeconomic backgrounds, and language barriers can easily affect the quality of care.^27,39,40^ Fisher et al. described the availability of seizure diaries in various languages for patients’ use^20^ could assist in overcoming language barriers or poor communication patterns that cause poor management of patients living with epilepsy.^38^ The researchers will ensure the final version of the new seizure diary is available in different languages (English, Afrikaans, Sesotho and Setswana) for patients and their caregivers.

Beghi, in a study about addressing the burden of epilepsy, reported on the views of patients, and caregivers of patients with epilepsy that adults with epilepsy had difficulties with marriage, driving and finding jobs in the labour market.^41^ Caregivers experienced increased stress, fear during seizures, depression, helplessness and joblessness because they have to care for the patients.^41^ Providing space for remarks in the seizure diary will enable patients and caregivers to express their views and describe the burden of epilepsy on them and their families so that healthcare workers reviewing the diary can better understand and find ways to assist them.

Participants who had previous exposure to a seizure diary had the opportunity to be exposed to both seizure diaries. Most of them preferred the new seizure diary to the old one because it contained more information. Blachut et al. stated that the provided information, if used for decision-making, strongly reinforces patients completing the diary and gives them treatment satisfaction.^36^ Most participants who chose the old seizure diary (Basic calendar diary) revealed that their reason for doing so was because it was easier to use. The basic calendar diary lacks the details that can assist healthcare professionals in making important clinical decisions. Fisher et al. described the information provided by the basic calendar diary as far from comprehensive in understanding longitudinal relationships in seizure events.^18^

Almost all participants agreed that the new seizure diary is useful for tracking patients’ seizures and providing information. This information is available for doctors and other healthcare workers to use in improving the quality of care they provide to their patients. Blachut et al. described seizure documentation as strongly related to doctors’ behaviours. If doctors take sufficient time to analyse and discuss the diary, it motivates the patients to continue to complete it because they know it can contribute to treatment decisions.^25,36^ This view has been supported by other studies about the seizure diary.^19,27^

The diary used during this study was designed based on recommendations from the Delphi expert panel^32^ and the recommendation of patients with epilepsy on what they think should be the content of a seizure diary.^22^ In addition to the recommendations of the Delphi experts, patients, relatives and caregivers of patients with epilepsy impacted by the disease actively participated and made recommendations they felt were important and would help motivate them to use the new diary. The changes recommended by participants from both groups in this study were incorporated into the diary to create the final version of the new seizure diary. This new seizure diary (Appendix 1) was created based on patient-centred care principles. Epstein and Street stated that patient-centred care should help patients know more about their health and facilitate taking ownership of their health.^42^

The final version of the new diary will be proposed to the Department of Health, seeking their permission to allow its distribution and use by patients with epilepsy.

### Recommendations

To improve the acceptance of the diary, the new seizure diary should be made available in different African languages for patients with epilepsy, their caregivers and relatives to use in their local dialect.

As smartphones, portable electronic devices and the cost of data become more affordable and accessible in our urban and rural areas. Future research should be geared towards partnering with international and local organisations to create an indigenous, culturally acceptable African Epilepsy mobile application for the use of patients living with epilepsy, relatives, caregivers and healthcare professional involved in their care. This will go a long way in improving the quality of care we provide for our patients and support for their families.

### Limitations of the study

This study was on patients from Kimberley and Bloemfontein, which may be subject to selection bias making it difficult to generalise the study’s findings. The change in clinic bookings to a 6-monthly schedule because of the COVID-19 pandemic impacted the ability to find and follow up with patients. The number of patients attending the clinics was significantly reduced during this time.

The focus of this study was only on the experience of the new seizure diary; the diary was checked to see whether the patients or caregivers were documenting the seizures in the diary, but we did not investigate the accuracy of the reported seizures. The feedback received from some participants was not informative; it lacked details.

## Conclusion

This study looked at the experience of the seizure diary by participants who used the diary for 6 months. The study included participants who were previously diary-unexposed and who previously used a seizure diary. The study provided valuable information about the experience of patients, caregivers and other family members from both groups while using the diary. Our findings show that all participants found the new seizure diary helped them to keep track of seizures. Most participants from both groups found the new seizure diary useful and were willing to continue using it.

## Figures and Tables

**FIGURE 1-A1:**
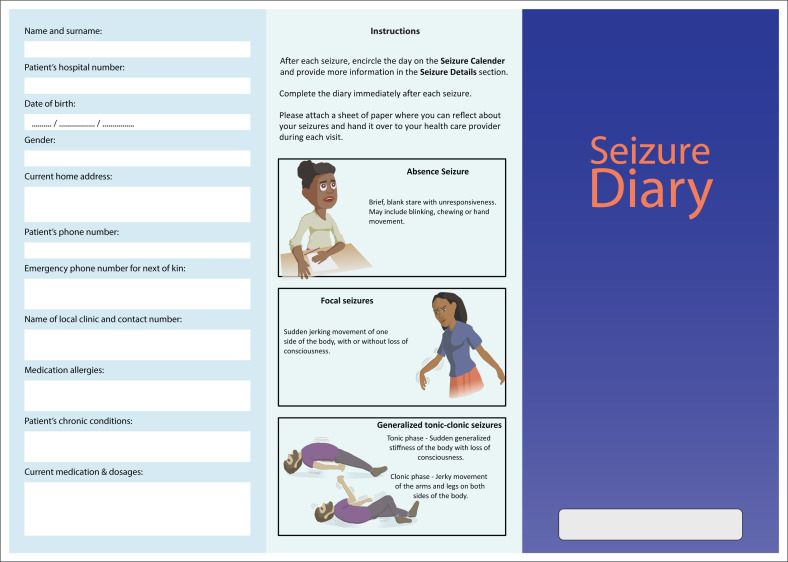
Seizure diary front.

**FIGURE 2-A1:**
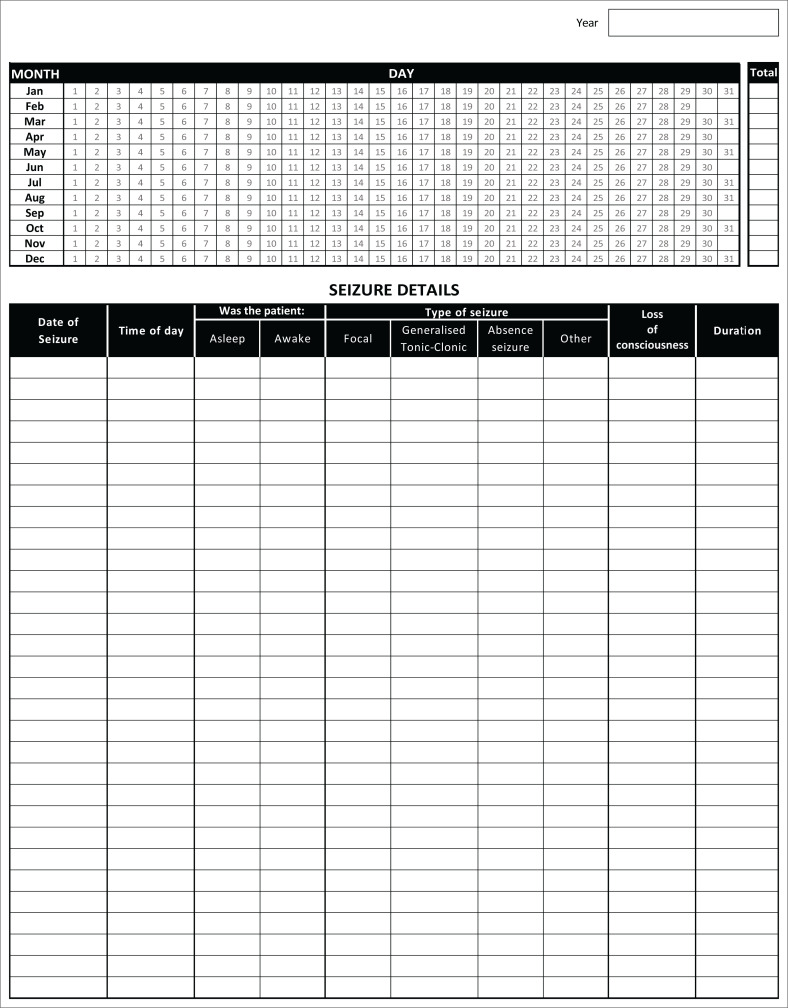
Seizure diary back.
